# Hydrolyzable vs. Condensed Wood Tannins for Bio-based Antioxidant Coatings: Superior Properties of Quebracho Tannins

**DOI:** 10.3390/antiox9090804

**Published:** 2020-08-31

**Authors:** Federica Moccia, Alessandra Piscitelli, Samuele Giovando, Paola Giardina, Lucia Panzella, Marco d’Ischia, Alessandra Napolitano

**Affiliations:** 1 Department of Chemical Sciences, University of Naples “Federico II”, Via Cintia 4, I-80126 Naples, Italy; federica.moccia@unina.it (F.M.); apiscite@unina.it (A.P.); giardina@unina.it (P.G.); dischia@unina.it (M.d.); alesnapo@unina.it (A.N.); 2 Centro Ricerche per la Chimica Fine Srl for Silvateam Spa, Via Torre 7, I-12080 San Michele Mondovì (CN), Italy; sgiovando@silvateam.com

**Keywords:** hydrolyzable tannins, condensed tannins, antioxidant, DPPH assay, FRAP assay, coating

## Abstract

Tannins have always been the subject of great interest for their countless properties, first of all their ability to produce functional coatings on a variety of materials. We report herein a comparative evaluation of the antioxidant properties of wood tannin-based coated substrates. In particular, nylon membrane filters were functionalized with chestnut (hydrolyzable) or quebracho (condensed) tannins by dip coating under different conditions. The efficiency of functionalization was evaluated by the 2,2-diphenyl-1-picrylhydrazyl (DPPH) and ferric reducing/antioxidant power (FRAP) assays, which invariably highlighted the superior ability of condensed tannins to induce the formation of a functional and robust coating. The results of the antioxidant assays revealed also the deleterious effects of aerial or enzymatic oxidation conditions on substrate functionalization, being more significant in the case of hydrolyzable tannins. On the other hand, the use of oxidizing conditions allowed to obtain more stable coatings, still exhibiting good antioxidant properties, in the case of condensed tannins. The presence of iron ions did not lead to a significant improvement of the coating efficiency for either tannins. The systematic approach used in this work provides novel and useful information for the optimal exploitation of tannins in antioxidant functional coatings.

## 1. Introduction

Tannins occupy a prominent role among natural phenolic compounds due to their remarkable antioxidant, antimicrobial, and anticancer activities [1,2,3]. Apart from these biologically relevant properties, these compounds have always been known for their ability to strongly interact with proteins, which has prompted their use in the leather tanning industry [4,5]. 

**Figure 1:**
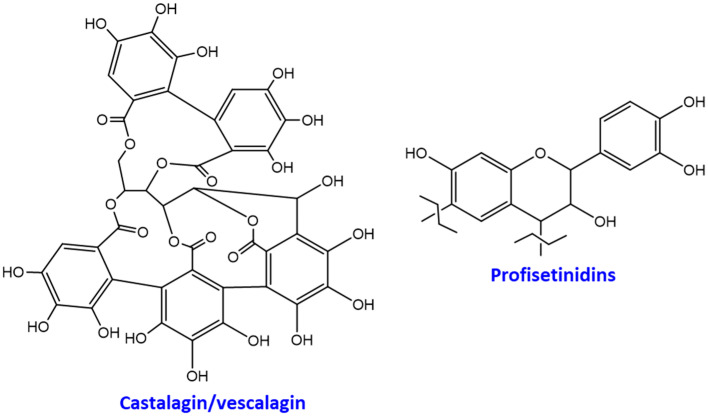
Representative structures of chestnut and quebracho tannins.

Tannins are widely distributed in the plant kingdom, but the most industrially exploited are those deriving from wood, particularly from *Castanea sativa* (chestnut) and *Shinopsis lorentzii* (quebracho) hardwood. These are traditionally classified as hydrolyzable or condensed (non-hydrolyzable) tannins. Chestnut tannins are composed mainly of hydrolyzable ellagitannins such as castalagin and its isomer vescalagin [6,7], whereas quebracho tannins comprise mainly condensed tannins of which linear profisetinidins represent the major constituents [8,9,10] (Figure 1). 

Wood tannins are currently used in food industry, e.g., as additives to improve wine aroma and taste, as crosslinkers to strengthen the network of vegetable-based food gelatins, as livestock food supplements to enhance meat quality, or as additives in antioxidant and antimicrobial active packaging [9,11,12,13]. 

Tannins are also currently exploited as adhesives or coatings for wood and other materials as well as crosslinkers in foams and resins, as metal corrosion inhibitors or as adsorbents in environmental applications [14,15,16,17,18]. 

In addition, several papers have reported the use of wood tannins for the implementation of functional films and coatings. As an example, condensed wood tannins from radiata pine bark have been used as protective coatings for acrylic-based surfaces [19], whereas condensed tannins from quebracho or acacia have been combined with cellulose nanofibrils for the development of hybrid, functional films [20,21,22]. Oak tannins [23,24] and chestnut tannins [25] have instead been used to enhance the antioxidant and antimicrobial properties of protein-based films. 

Although it is now well-established that coating properties of wood tannins are to be ascribed to their ability to interact with macromolecules by hydrogen bondings and hydrophobic interactions [26,27], a comparative investigation between hydrolyzable and condensed wood tannins for the development of functional antioxidant coatings is, to the best of our knowledge, lacking in the literature.

On this basis we report herein a comparative evaluation of the antioxidant properties of substrates coated with chestnut and quebracho wood tannins. 

## 2. Materials and Methods

### 2.1. General Experimental Methods

Chestnut tannins (CT) and quebracho tannins (QT) were provided by Silvateam (S. Michele Mondovì, Cuneo, Italy).

Whatman^®^ Nylon membrane filters (0.45 μm pore size, 47 mm diameter) were from Sigma-Aldrich (Milan, Italy). 

2,2-Diphenyl-1-picrylhydrazyl (DPPH), iron (III) chloride hexahydrate (97%), iron (II) chloride tetrahydrate, iron (II) sulfate, 2,4,6-tris(2-pirydyl)-s-triazine (TPTZ) (≥98%), (±)-6-hydroxy-2,5,7,8-tetramethylchromane-2-carboxylic acid (Trolox) (97%), sodium borohydride, and ethylenediaminetetraacetic acid (EDTA) sodium salt were obtained from Sigma-Aldrich (Milan, Italy) and used as obtained. 

The laccase used in this study was the recombinant POXA1b from the fungus *Pleurotus ostreatus* heterologously produced in the yeast *Pichia pastoris* [28].

UV-Vis spectra were recorded using a HewlettPackard 8453 Agilent spectrophotometer. 

Chemical structures were drawn with the software ChemDraw Ultra 12.0.

### 2.2. Coating of Nylon Membrane Filters with Tannins

For each tannin, the following solutions were prepared:

- 7.5 mg of tannin dissolved in 15, 75 or 375 mL of distilled water
- 3.0 mg of tannin dissolved in 30 mL of distilled water containing 300 µL of a 1.7 U/mL laccase solution
- 3.0 mg of tannin dissolved in 30 mL of 0.05 M phosphate buffer (pH 6.0) containing 300 µL of a 1.7 U/mL laccase solution
- 3.0 mg of tannin dissolved in 30 mL of distilled water containing 3.5 mg of FeSO_4_
- 7.5 mg of tannin dissolved in 75 mL of 0.05 M carbonate buffer (pH 9.0)

When necessary, the solutions were kept in an ultrasonic bath for 15 min to promote tannin dissolution. To check for this, UV-vis spectra of the tannin solutions were recorded before and after centrifugation (7000 rpm, 30 min, 25 °C).

The nylon filters were dipped in each solution and the mixtures were kept under magnetic stirring. After 2 h the filters were removed from the solution, abundantly washed with double-distilled water and allowed to air dry. 

In another series of experiments, the different reaction mixtures were periodically analyzed by UV-vis spectroscopy. When necessary, after 2 h of incubation the mixtures were treated as follows and analyzed again by UV-vis spectroscopy:

(1) addition of 6 M HCl until pH 2;
(2) addition of NaBH_4_ followed or not by acidification with 6 M HCl;
(3) addition of EDTA (sodium salt).

### 2.3. DPPH Assay

Nylon filters (5 mg) were added to 5 mL of a 200 µM ethanolic solution of DPPH [29,30]. The samples were kept at room temperature and each solution was spectrophotometrically analyzed at 515 nm after 10 min and 2.5 h. The assay was carried out on the filters as such and previously washed with ethanol, under the same conditions used for the assay.

In other experiments, the DPPH assay was carried out on an aqueous solution of CT or QT (20 µL of a 0.5 mg/mL solution added to 2 mL of DPPH solution). Experiments were run in triplicate.

### 2.4. Ferric Reducing/Antioxidant Power (FRAP) Assay 

Nylon filters (5 mg), previously washed with 0.3 M acetate buffer at pH 3.6 for 2.5 h, were added to 2 mL of a freshly prepared solution of 1.7 mM FeCl_3_ and 0.83 mM TPTZ in 0.3 M acetate buffer (pH 3.6) at 10:1:1 *v*/*v*/*v* ratio [31]. The mixtures were kept at room temperature and the absorbance at 593 nm was measured after 10 min and after 2.5 h. 

In other experiments, the assay was carried out on an aqueous solution of CT or QT (20 µL of a 0.1 mg/mL solution added to 2 mL of FRAP solution). Trolox was used as the reference antioxidant. Experiments were run in triplicate.

### 2.5. Quantification of Tannin Deposition on the Nylon Membrane Filters 

Nylon filters (8 mg) were dipped in freshly prepared solutions (8 mL) of:

- 0.5, 0.1, and 0.02 mg/mL QT or CT in distilled water
- 0.1 mg/mL QT or CT in distilled water containing 300 µL of a 1.7 U/mL laccase solution
- 0.1 mg/mL QT or CT in 0.05 M phosphate buffer (pH 6.0) containing 300 µL of a 1.7 U/mL laccase solution
- 0.1 mg/mL QT or CT in 0.05 M carbonate buffer (pH 9.0).

The mixtures were kept under magnetic stirring at room temperature and after 2 h the UV-vis spectra of the solutions were recorded. The amount of tannin deposited on the nylon filter was evaluated by determining the decrease in absorbance at 280 nm for QT and 270 nm for CT in comparison to control solutions not containing the filters.

### 2.6. Statistical Analysis

All the antioxidant experiments were run at least in triplicate. The results are presented as mean ± SD values and compared by Microsoft Excel Student’s *t*-test (2019 version).

## 3. Results and Discussion

### 3.1. Coating of Nylon Membrane Filters with Tannins

**Figure 2:**
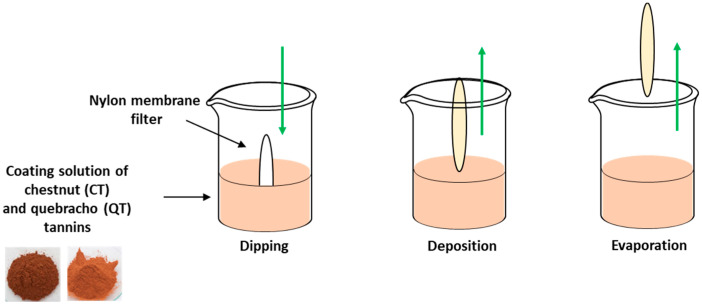
Schematic representation of the dip coating technique for thin film deposition.

Nylon membrane filters were chosen as the substrates to be functionalized based on their highly homogeneous and uniform polymeric network and the remarkable affinity of tannins toward polyacrylamide [32]. Functionalization was achieved by a simple dip coating procedure, involving immersion of the substrate into a solution of the coating material (Figure 2).

Several conditions were adopted in order to explore the effects of different parameters on substrate functionalization, that is tannin concentration, the use of oxidizing conditions, and presence of metal ions. 

In particular, nylon membrane filters were dipped into aqueous solutions of CT or QT at three different concentrations (0.02, 0.1, and 0.5 mg/mL), the highest one corresponding to the maximum solubility of tannins in water, and after 2 h they were withdrawn, extensively washed with water, and left to dry overnight. 

The use of oxidizing conditions is generally reported as fundamental to achieve efficient coatings with phenolic compounds such as dopamine, the precursor of polydopamine (PDA) that is universally regarded as the reference material for surface functionalization [33,34,35]. In this work two different oxidizing systems were taken in consideration, that is the enzyme laccase (in water or in phosphate buffer at pH 6.0) or a 0.05 M carbonate buffer at pH 9.0, which is the medium widely reported in literature to implement PDA coatings [36,37].

Finally, the effects of iron ions in promoting functional coating formation was evaluated. Iron(II) ions are commonly employed as a fundamental ingredient in the widespread used iron gall ink [15,38,39] and iron-polyphenol coordination has been emerging as a highly promising tool for design and synthesis of functional materials [40]. 

**Figure 3:**
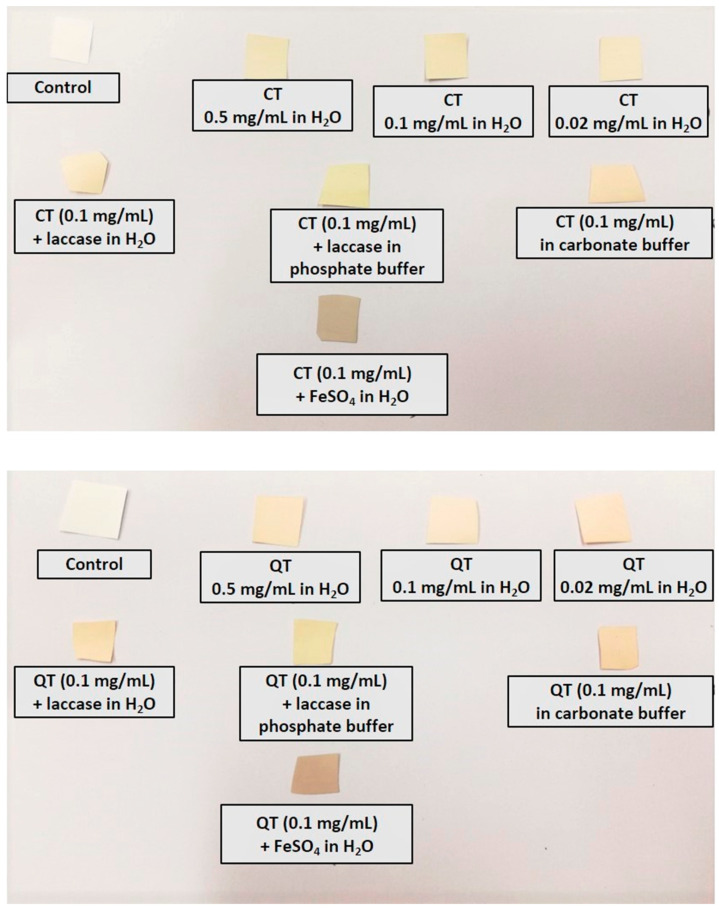
Nylon membrane filters coated with chestnut tannins (CT) (**top**) and quebracho tannins (QT) (**bottom**) under the different dip coating conditions explored.

Figure 3 shows the colors acquired by the nylon filters under the different dip coating conditions, which appeared more intense when oxidizing agents or iron ions were used. 

### 3.2. Antioxidant Properties of Tannin-Coated Nylon Membrane Filters

#### 3.2.1. DPPH Assay

The percentage of DPPH reduction by the various nylon membrane filters at different times are reported in Table 1.

As expected, a progressive increase in DPPH reduction was observed over time, and the differences in the effectiveness of the samples tended to flatten out passing from 10 min to 2 h 30 min. Generally, higher DPPH reducing properties were determined for QT- compared to CT-coated membrane filters. Moreover, higher concentrations of tannin led to materials endowed with higher antioxidant functionality, likely as a result of a more efficient coating. Remarkably lower antioxidant properties were measured when oxidizing conditions, e.g., aerial oxidation in carbonate buffer, were adopted, especially in the case of CT, which on the contrary apparently benefited from the presence of iron ions. 

With the aim to determine whether the differences in the antioxidant properties observed are to be ascribed to differences in the efficiency of the coating process or to structural modifications occurring on tannins under the different experimental conditions adopted, in a separate series of experiments the amount of tannin deposited on the nylon filter was evaluated by UV-vis spectroscopy. As shown in Figure S1 for the 0.5 mg CT and QT solutions as representative examples, a higher decrease of absorbance was observed after 2 h for the solutions containing the nylon filter compared to control mixtures not containing the substrate, likely as a consequence of efficient material coating by the polyphenolic samples. The amounts of tannins adhered to the nylon membrane filter under the different coating conditions are reported in Table S1. As expected, the most efficient coating was achieved with the tannin solutions at the highest concentrations, that is 0.5 mg/mL, in line with the results of the DPPH assay, although no linear correlation was observed between the amount of tannin adhered and the concentration of the initial solutions either for QT or CT (Figure S2). The use of oxidizing conditions, particularly alkaline carbonate buffer, invariably led to deposition of lower amounts of material, in line again with the results of the DPPH assay. However, weak or no linear correlations were observed between the percentage of DPPH reduced and the amount of tannin adhered to the nylon filter (Figure S3), suggesting that it is not simply the amount of coating material to determine the efficacy of the functionalized substrate as an antioxidant, but likely also the chemical structure of the coating species. 

To check if DPPH reduction was due to a solubilization and hence a release of tannins or tannin-derived species into the organic solvent used for the assay, in subsequent experiments the coated substrates were taken in ethanol under the same conditions used for the DPPH assay and after 2.5 h the UV-vis spectra of the solutions were recorded. As shown in Figure S4, variable amounts of tannins depending on the coating conditions appeared to be released in ethanol, although no qualitative differences among the solubilized species were evident. Accordingly, the DPPH assay was repeated on the coated filters after being washed with ethanol. 

**Figure 4:**
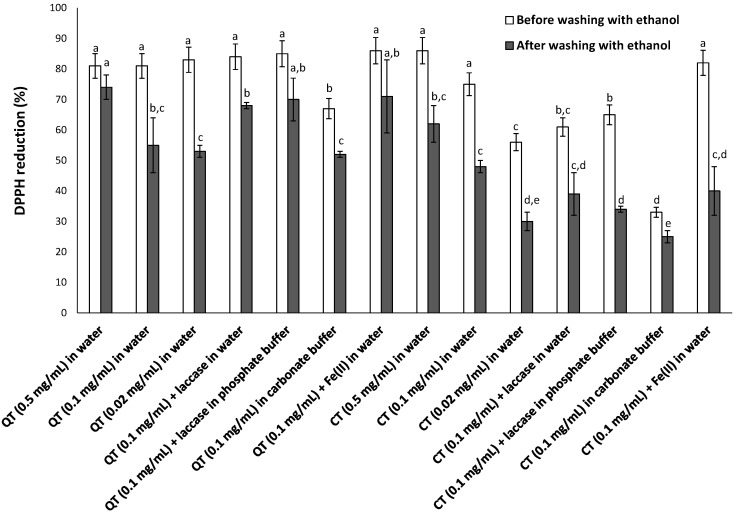
DPPH reduction at 2.5 h by the tannin-coated nylon membrane filters before and after washing with ethanol. Reported are the mean ± SD values from at least three experiments. Values without a common letter are significantly different (*p* < 0.05).

As expected, lower antioxidant capacities were exhibited by the washed substrates, although a rather high percentage of DPPH reduction was still observed, especially when QT were used to coat the filters (Figure 4). The detrimental effect of washing was particularly evident when the dip coating was performed in water, whereas the employment of oxidizing conditions (laccase or alkaline pH) led to more robust coatings, especially in the case of QT. Less significant were the effects induced by iron ions on the antioxidant properties of QT- and CT-coated membrane filters, although the latter were again more susceptible to ethanol washing. 

To investigate if in general the differences observed in the antioxidant properties of the substrates coated under the different conditions, especially regarding those obtained in water, were due to intrinsic differences in the antioxidant properties of CT and QT or rather to a different coating efficiency, in other experiments the DPPH assay was performed on aqueous solutions of the two tannin samples (Table 2). In agreement with what was previously reported [41], at the same weight the highest antioxidant activity was recorded for the CT sample. This is in contrast with the results of the DPPH assay run on the coated membrane filters, therefore suggesting a higher efficiency of coatings with QT or QT-deriving species.

#### 3.2.2. FRAP Assay

**Figure 5:**
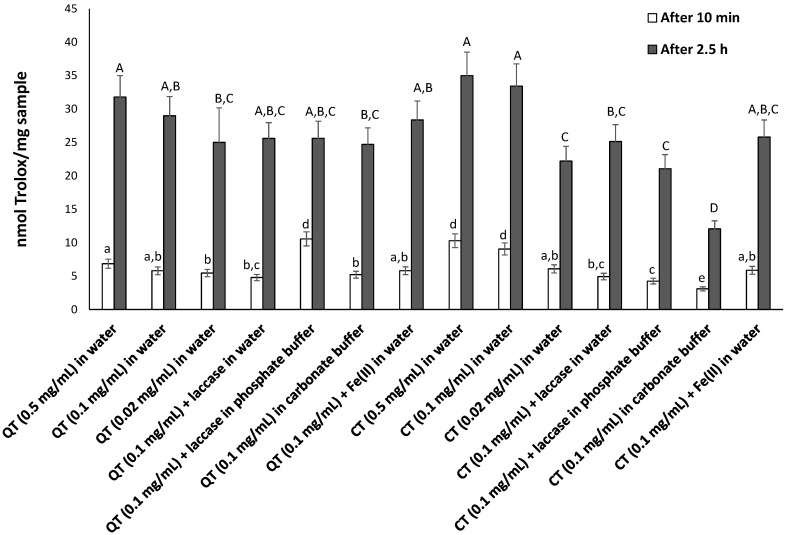
Trolox eqs determined at 10 min and 2.5 h for the tannin-coated nylon membrane filters in the ferric reducing/antioxidant power (FRAP) assay. Reported are the mean ± SD values from at least three experiments. Values in a series without common letters are significantly different (*p* < 0.05).

In other experiments the antioxidant properties of the nylon membrane filters coated with CT and QT under the different conditions explored were evaluated in aqueous medium by the FRAP assay. Based on what observed before, the experiments were performed directly on filters preliminarily washed with the assay medium, that is 0.3 M acetate buffer (pH 3.6), although in this case no significant release of the coating tannins was observed (UV-vis analysis, not shown). The Trolox equivalents determined for each coated substrate are reported in Table S2 and Figure 5. Compared to the DPPH assay, the differences between the CT- and QT-functionalized substrates were less pronounced, although also in this case lower reducing properties were exhibited by the CT-coated materials produced under oxidizing conditions, especially alkaline pH. Moreover, also in the FRAP assay CT were found to be more active than QT (Table 2), confirming the superior coating ability of this latter especially under oxidizing conditions. In any case no strong correlation was again observed between the amount of tannin deposited on the filter and the antioxidant properties exhibited by the coated substrates (Figure S5).

### 3.3. Spectrophotometric Analysis of Tannin Solutions

**Figure 6:**
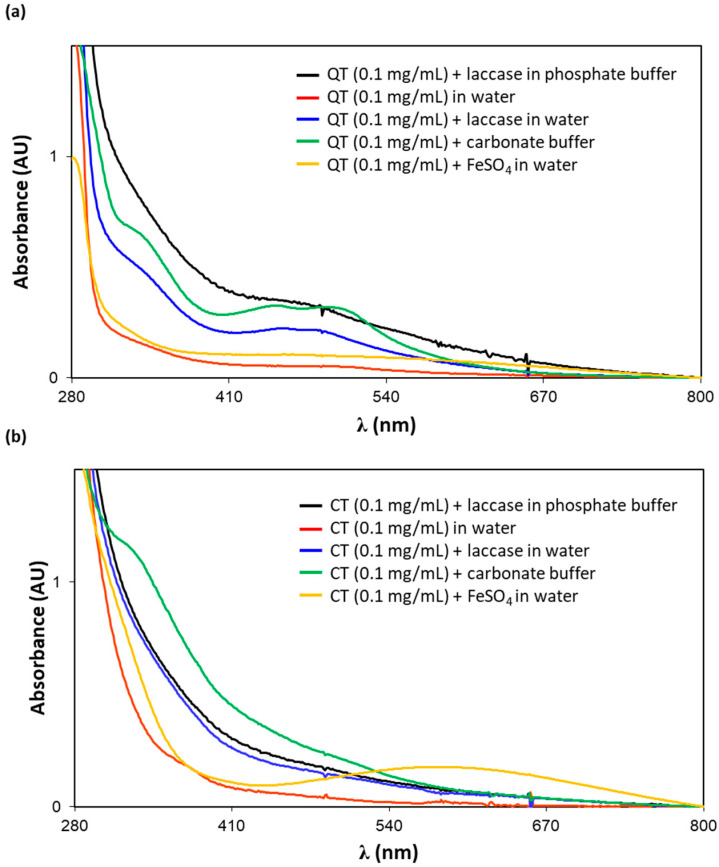
UV-vis spectra of the dip coating solutions at 2 h. (**a**) QT; (**b**) CT.

With a view to obtaining information on the chemical modifications occurring on CT and QT under the different dip coating conditions, the various solutions were periodically analyzed by UV-vis spectroscopy. Figure 6 shows the UV-vis spectra of the different dip coating solutions recorded at 2 h, that is the time at which the nylon filter was removed. When laccase or alkaline pH was employed, a more or less defined chromophore appeared in the visible region for both tannins, likely suggesting formation of oxidation products. Completely different chromophoric species were instead detected in the presence of iron ions, as particularly evident in the case of CT. 

To further characterize the species produced under the different conditions, in other experiments a series of additives were added to the solutions at 2 h reaction time, that is the same time employed in the nylon filter coating procedure. The formation of oxidation products under alkaline conditions was supported by the disappearance of the visible chromophore following addition of sodium borohydride (Figure S6). The same applies to the solution containing laccase, although in this case the changes in the UV-vis spectra were less marked (Figure S7). On the contrary, the involvement of oxidative pathways was ruled out when iron ions were used, since the visible chromophore generated under these conditions was not affected by the addition of reducing agents (Figure S8). In this case, formation of a tannin/iron complex could be reasonably envisaged, in agreement with the disappearance of the visible chromophore induced by acidification. This complex seemed also to be fairly stable, given its apparent resistance to treatment with strong chelating agent such as EDTA, which even in excess was able to induce only a partial loss of the chromophore. To what extent the chemical transformations observed are relevant to the different antioxidant properties exhibited by the tannin-coated nylon filters is an aspect which is currently under investigation. 

## 4. Conclusions

The potential use of wood tannins for film deposition and functional antioxidant coatings has been investigated by comparing representative members of the condensed and hydrolyzable variants. The most relevant findings and observations from this study can be summarized as follows.

First, condensed tannins, e.g., quebracho tannins, generated coatings with a superior antioxidant ability.

Second, although associated with a decrease in the antioxidant properties, oxidative dip coating conditions proved more efficient to produce robust films with a lower tendency to leaching, as a consequence of polymerization processes.

Third, with both tannins the coating efficiency was not affected by the presence of iron ions which are commonly reported additives in film deposition protocols.

Although still preliminary given the relatively low number of substrates and functional properties explored, this study provides interesting and useful data for a full exploitation of tannins in antioxidant functional coatings.

## Figures and Tables

**Table 1:** 2,2-Diphenyl-1-picrylhydrazyl (DPPH) reduction at different times by the tannin-coated nylon membrane filters. ^1^

Sample	DPPH Reduced (%) (after 10 min)	DPPH Reduced (%) (after 2.5 h)
QT (0.5 mg/mL) in H_2_O	85 ± 6 ^a^	81 ± 3 ^a^
QT (0.1 mg/mL) in H_2_O	83 ± 4 ^a^	81 ± 2 ^a^
QT (0.02 mg/mL) in H_2_O	56 ± 3 ^b^	83 ± 4 ^a^
QT (0.1 mg/mL) + laccase in H_2_O	79 ± 4 ^a^	84 ± 5 ^a^
QT (0.1 mg/mL) + laccase in phosphate buffer (pH 6.0)	61 ± 3 ^b^	85 ± 6 ^a^
QT (0.1 mg/mL) in carbonate buffer (pH 9.0)	41 ± 2 ^c^	67 ± 4 ^b^
QT (0.1 mg/mL) + FeSO_4_ in H_2_O	76 ± 4 ^a^	86 ± 6 ^a^
CT (0.5 mg/mL) in H_2_O	56 ± 3 ^b^	86 ± 6 ^a^
CT (0.1 mg/mL) in H_2_O	23 ± 1 ^d^	75 ± 3 ^a^
CT (0.02 mg/mL) in H_2_O	28 ± 1 ^e^	56 ± 3 ^c^
CT (0.1 mg/mL) + laccase in H_2_O	25 ± 1 ^d^	61 ± 3 ^b,c^
CT (0.1 mg/mL) + laccase in phosphate buffer (pH 6.0)	25 ± 1 ^d^	65 ± 4 ^b^
CT (0.1 mg/mL) in carbonate buffer (pH 9.0)	7 ± 1 ^f^	33 ± 2 ^d^
CT (0.1 mg/mL) + FeSO_4_ in H_2_O	40 ± 2 ^c^	82 ± 4 ^a^

^1^ Reported are the mean ± SD values of at least three experiments. Values in a column without a common letter (^a–f^) are significantly different (*p* < 0.05).

**Table 2:** Antioxidant properties of CT and QT. ^1^

Sample	DPPH Reduced (%) ^2^ (after 2.5 h)	μmol Trolox/mg Sample (FRAP Assay) (after 2.5 h)
QT	37 ± 2 ^a^	6.1 ± 0.3 ^a^
CT	47 ± 3 ^b^	7.0 ± 0.4 ^b^

^1^ Reported are the mean ± SD values of at least three experiments. Values in a column with different letters (^a,b^) are significantly different (*p* < 0.05). ^2^ Data for a 0.005 mg/mL tannin solution.

## Supplementary Materials

The following are available online at https://www.mdpi.com/2076-3921/9/9/804/s1, Figure S1. Comparison of the UV-vis spectra of tannin solutions in the presence or absence of the nylon filters; Table S1. Amounts of tannins adhered to the nylon membrane filters under the different coating conditions; Figure S2. Correlation between the amounts of tannin adhered to the nylon membrane filters and the concentration of the initial solutions of QT and CT; Figure S3. Correlation between the percentage of DPPH reduced by the functionalized substrates and the amounts of tannin adhered; Figure S4. UV-vis spectra of released tannins from the nylon membrane filters after washing in ethanol; Table S2. Trolox equivalents determined for the tannin-coated nylon membrane filters in the FRAP assay; Figure S5. Correlation between the Fe^3+^-reducing properties of the functionalized substrates and the amounts of tannin adhered; Figure S6. UV-vis spectra of tannin solutions in 0.1 M carbonate buffer (pH 9.0), before and after addition of different additives; Figure S7. UV-vis spectra of tannin and laccase solutions in 0.05 M phosphate buffer (pH 6.0), before and after addition of different additives, Figure S8. UV-vis spectra of tannin and FeSO_4_ solutions in water, before and after addition of different additives.
