# Supplementary material for: Hydrolyzable vs. Condensed Wood Tannins for Bio-based Antioxidant Coatings: Superior Properties of Quebracho Tannins
Source: Antioxidants (Basel). 2020 Aug 31;9(9):804. doi: 10.3390/antiox9090804 (PMC7556001; doi:10.3390/antiox9090804)
Supplement: Supplementary file 1 [file antioxidants-09-00804-s001.pdf]

Supplementary Materials

Hydrolyzable *vs.* Condensed Wood Tannins for Bio-based Antioxidant Coatings

Federica Moccia [1], Alessandra Piscitelli [1], Samuele Giovando [2], Paola Giardina [1], Lucia Panzella [1,*], Marco d'Ischia [1] and Alessandra Napolitano [1]

1 Department of Chemical Sciences, University of Naples "Federico II", Via Cintia 4, I-80126 Naples, Italy; federica.moccia@unina.it (F.M.); apiscite@unina.it (A.P.); giardina@unina.it (P.G.); dischia@unina.it (M.d.I.); alesnapo@unina.it (A.N.)
2 Centro Ricerche per la Chimica Fine Srl for Silvateam Spa, Via Torre 7, I-12080, San Michele Mondovì, CN, Italy; sgiovando@silvateam.com
* Correspondence: panzella@unina.it; Tel.: +39-081-674131

Table of contents **Page**

Figure S1. Comparison of the UV-vis spectra at 2 h of 0.5 mg/mL (a) QT and (b) CT solutions in the presence or absence of the nylon filter. 2

Table S1. Amounts of tannins adhered to the nylon membrane filters under the different coating conditions. 3

Figure S2. Correlation between the amounts of tannin adhered to the nylon membrane filters and the initial concentrations of (a) QT and (b) CT in water . 4

Figure S3. Correlation between the percentages of DPPH reduced by the functionalized substrates and the amounts of tannin adhered. 5

Figure S4. UV-vis spectra of (a) released QT and (b) released CT from the nylon filters after washing in ethanol for 2.5 h. 6

Table S2. Trolox equivalents determined for the tannin-coated nylon membrane filters in the FRAP assay. 7

Figure S5. Correlation between the Fe^{3+}-reducing properties of the functionalized substrates and the amounts of tannin adhered. 8

Figure S6. UV-vis spectra of (a) QT and (b) CT solutions in 0.1 M carbonate buffer (pH 9.0), before and after addition of different additives. 9

Figure S7. UV-vis spectra of (a) QT and (b) CT solutions in 0.05 M phosphate buffer (pH 6.0) containing laccase, before and after addition of different additives. 10

Figure S8. UV-vis spectra of (a) QT and (b) CT solutions in water containing $FeSO_4$, before and after addition of different additives. 11

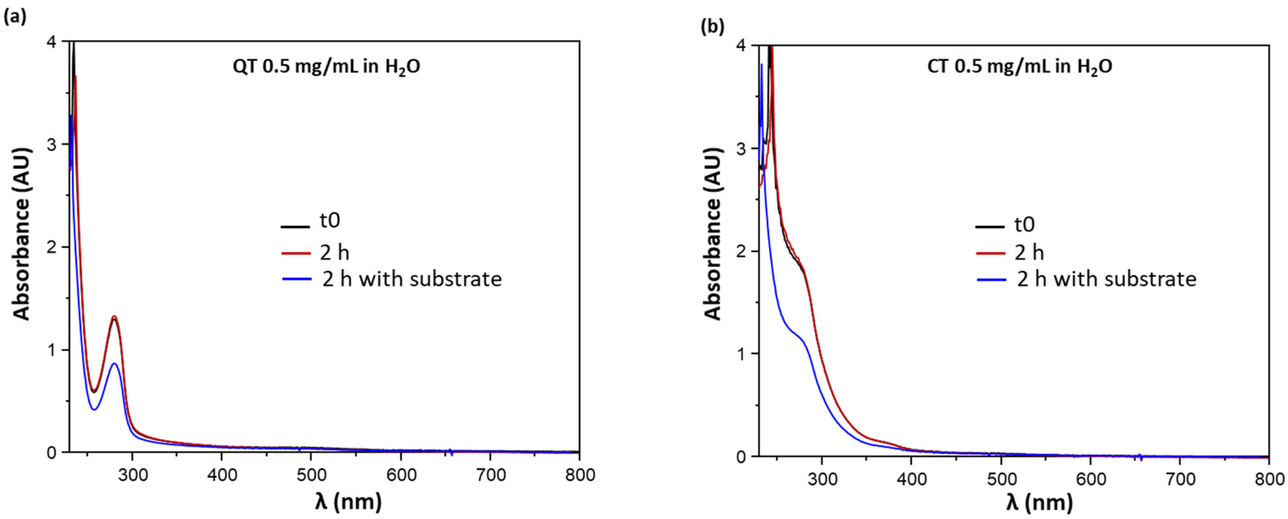

Figure S1. Comparison of the UV-vis spectra at 2 h of 0.5 mg/mL **(a)** QT and **(b)** CT solutions in the presence or absence of the nylon filter.

Table S1. Amounts of tannin adhered to the nylon membrane filter under the different coating conditions.[1]

Sample	mg of tannins adsorbed/mg substrate (after 2 h)
QT (0.5 mg/mL) in H_2O	0.28 ± 0.01
QT (0.1 mg/mL) in H_2O	0.031 ± 0.001
QT (0.02 mg/mL) in H_2O	0.0061 ± 0.0003
QT (0.1 mg/mL) + laccase in H_2O	0.031 ± 0.002
QT (0.1 mg/mL) + laccase in phosphate buffer pH 6.0	0.021 ± 0.001
QT (0.1 mg/mL) in carbonate buffer pH 9.0	0.021 ± 0.001
CT (0.5 mg/mL) in H_2O	0.26 ± 0.01
CT (0.1 mg/mL) in H_2O	0.042 ± 0.002
CT (0.02 mg/mL) in H_2O	0.041 ± 0.002
CT (0.1 mg/mL) + laccase in H_2O	0.041 ± 0.002
CT (0.1 mg/mL) + laccase in phosphate buffer pH 6.0	0.032 ± 0.002
CT (0.1 mg/mL) in carbonate buffer pH 9.0	0.011 ± 0.001

[1] Reported are the mean ± SD values of at least three experiments.

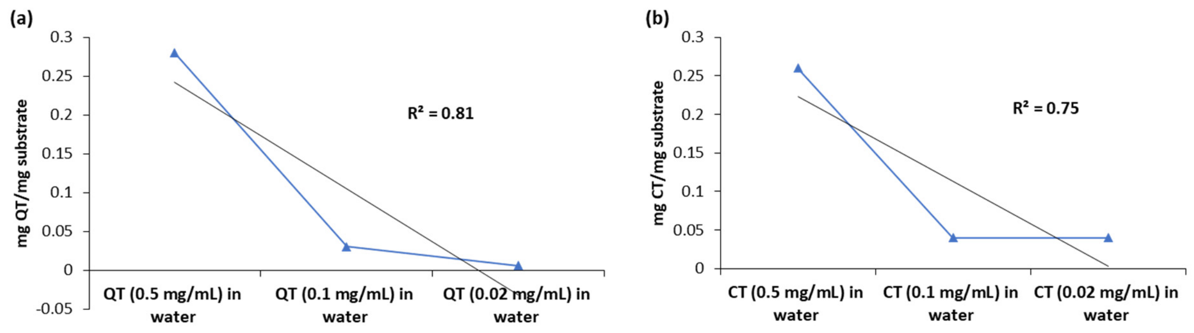

Figure S2. Correlation between the amounts of tannin adhered to the nylon membrane filters and the initial concentrations of (a) QT and (b) CT in water.

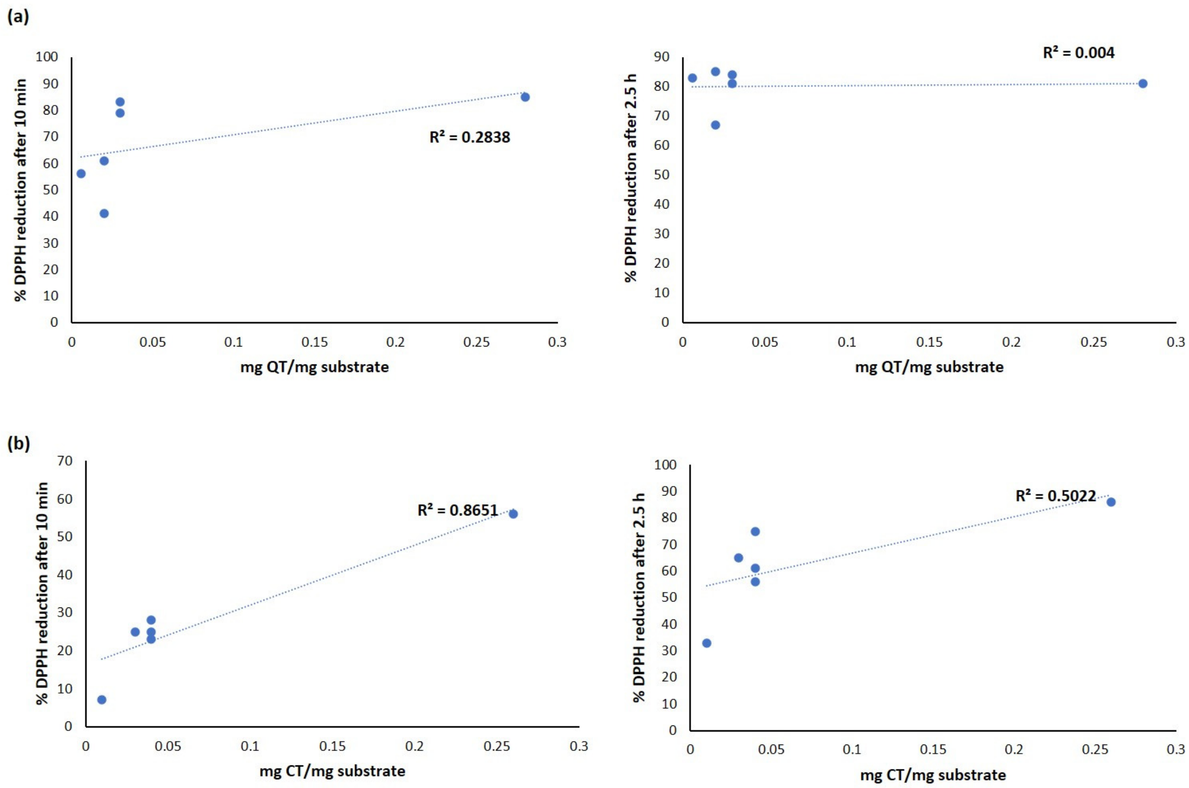

Figure S3. Correlation between the percentages of DPPH reduced after 10 min (left) and 2.5 h (right) and the amounts of **(a)** QT and **(b)** CT adhered to the nylon membrane filters.

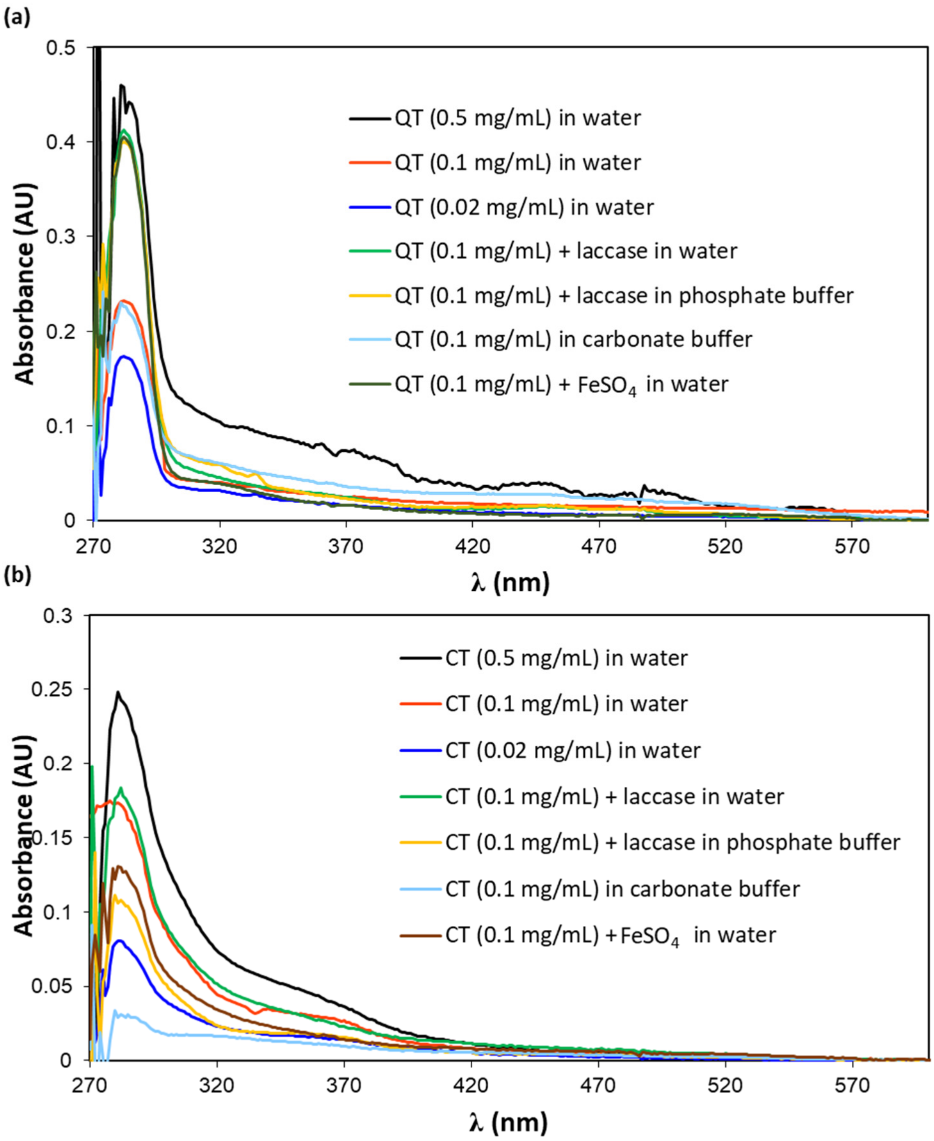

Figure S4. UV-vis spectra of (**a**) released QT and (**b**) released CT from the nylon filters after washing in ethanol for 2.5 h.

Table S2. Trolox equivalents determined for the tannin-coated nylon membrane filters in the FRAP assay.[1.]

Sample	nmol Trolox/mg filter (after 10 min)	nmol Trolox/mg filter (after 2.5 h)
QT (0.5 mg/mL) in H_2O	6.9 ± 0.7[a]	32 ± 3[a]
QT (0.1 mg/mL) in H_2O	5.8 ± 0.6[a,b]	29 ± 3[a,b]
QT (0.02 mg/mL) in H_2O	5.5 ± 0.5[b]	25 ± 2[b,c]
QT (0.1 mg/mL) + laccase in H_2O	4.8 ± 0.5[b,c]	26 ± 3[a,b,c]
QT (0.1 mg/mL) + laccase in phosphate buffer (pH 6.0)	11 ± 1[d]	26 ± 3[a,b,c]
QT (0.1 mg/mL) in carbonate buffer (pH 9.0)	5.2 ± 0.5[b]	25 ± 2[b,c]
QT (0.1 mg/mL) + $FeSO_4$ in H_2O	5.8 ± 0.6[a,b]	28 ± 3 [a,b]
CT (0.5 mg/mL) in H_2O	10 ± 1[d]	35 ± 4[a]
CT (0.1 mg/mL) in H_2O	9 ± 1[d]	33 ± 3[a]
CT (0.02 mg/mL) in H_2O	6.1 ±0.7[a,b]	22 ± 2[c]
CT (0.1 mg/mL) + laccase in H_2O	4.9 ± 0.5[b,c]	25 ± 3[b,c]
CT (0.1 mg/mL) + laccase in phosphate buffer (pH 6.0)	4.2 ± 0.4[c]	21 ± 2[c]
CT (0.1 mg/mL) in carbonate buffer (pH 9.0)	3.1 ±0.3[e]	12 ± 1[d]
CT (0.1 mg/mL) + $FeSO_4$ in H_2O	5.9 ±0.6[a,b]	26 ± 3[a,b,c]

[1] Reported are the mean ± SD values of at least three experiments. Values in a column without a common letter are significantly different ($P < 0.05$).

(a)

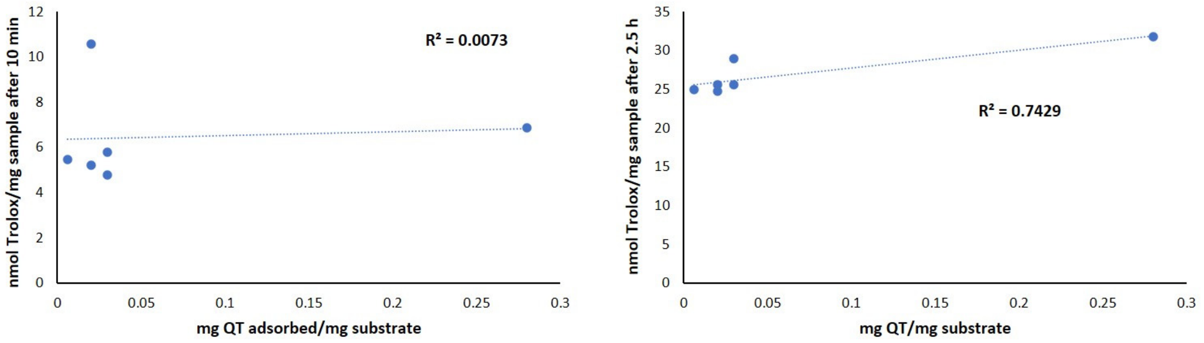

(b)

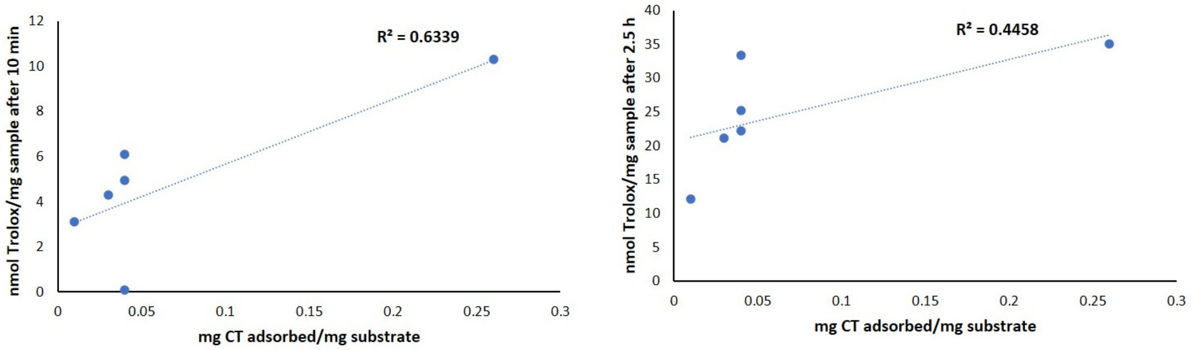

Figure S5. Correlation between the Fe^{3+}-reducing properties of the functionalized substrates after 10 min (left) and 2.5 h (right) and the amounts of **(a)** QT and **(b)** CT adhered to the substrate.

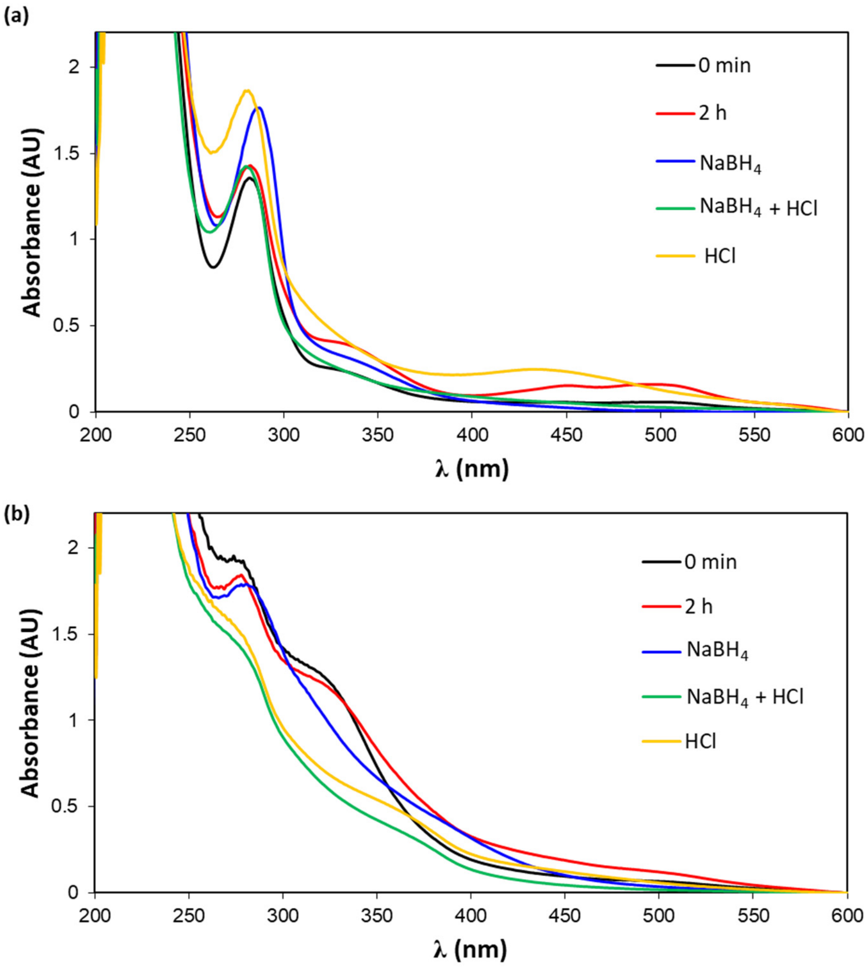

Figure S6. UV-vis spectra of (**a**) QT and (**b**) CT solutions in 0.1 M carbonate buffer (pH 9.0), before and after addition of different additives.

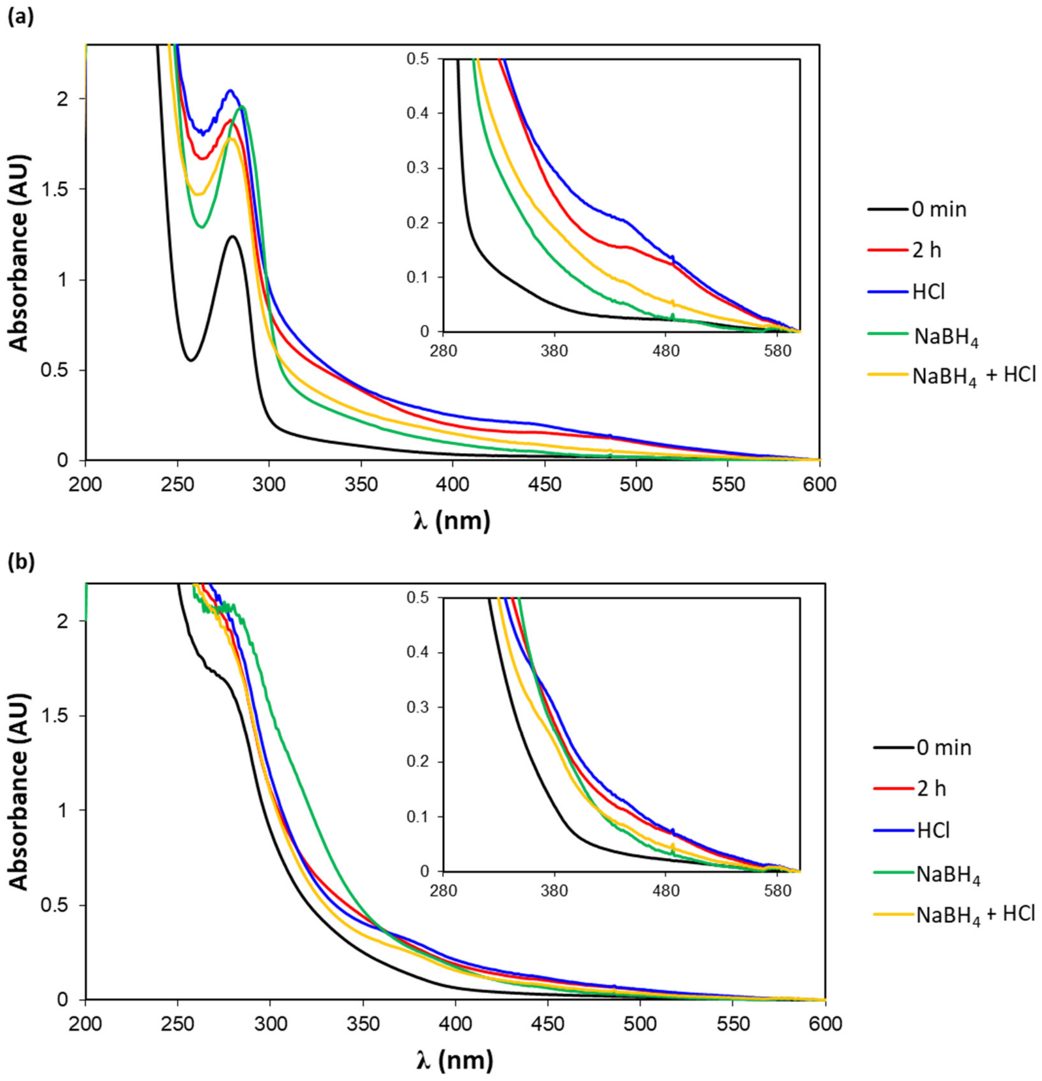

Figure S7. UV-vis spectra of (**a**) QT and (**b**) CT solutions in 0.05 M phosphate buffer (pH 6.0) containing laccase, before and after addition of different additives.

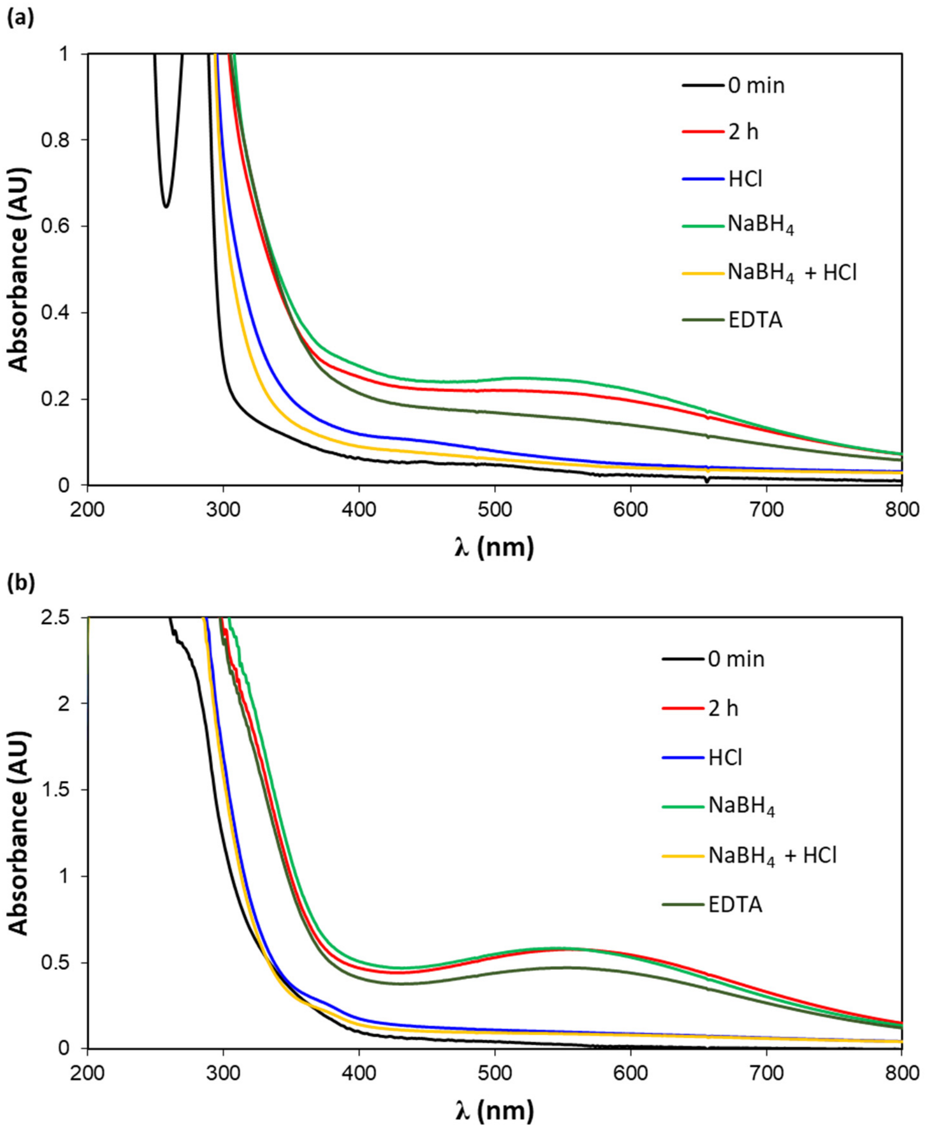

Figure S8. UV-vis spectra of (**a**) QT and (**b**) CT solutions in water containing $FeSO_4$, before and after addition of different additives.
